# Update on Thiamine Triphosphorylated Derivatives and Metabolizing Enzymatic Complexes

**DOI:** 10.3390/biom11111645

**Published:** 2021-11-07

**Authors:** Lucien Bettendorff

**Affiliations:** Laboratory of Neurophysiology, GIGA Neurosciences, University of Liège, 4000 Liège, Belgium; L.Bettendorff@uliege.be; Tel.: +32-4-366-5967

**Keywords:** thiamine triphosphate, adenosine thiamine triphosphate, thiamine triphosphatase, adenylate kinase, CYTH, triphosphate tunnel metalloenzymes, thiamine diphosphokinase, ATP synthase, thiamine diphosphate, glutamate dehydrogenase

## Abstract

While the cellular functions of the coenzyme thiamine (vitamin B1) diphosphate (ThDP) are well characterized, the triphosphorylated thiamine derivatives, thiamine triphosphate (ThTP) and adenosine thiamine triphosphate (AThTP), still represent an intriguing mystery. They are present, generally in small amounts, in nearly all organisms, bacteria, fungi, plants, and animals. The synthesis of ThTP seems to require ATP synthase by a mechanism similar to ATP synthesis. In *E. coli*, ThTP is synthesized during amino acid starvation, while in plants, its synthesis is dependent on photosynthetic processes. In *E. coli*, ThTP synthesis probably requires oxidation of pyruvate and may play a role at the interface between energy and amino acid metabolism. In animal cells, no mechanism of regulation is known. Cytosolic ThTP levels are controlled by a highly specific cytosolic thiamine triphosphatase (ThTPase), coded by *thtpa*, and belonging to the ubiquitous family of the triphosphate tunnel metalloenzymes (TTMs). While members of this protein family are found in nearly all living organisms, where they bind organic and inorganic triphosphates, ThTPase activity seems to be restricted to animals. In mammals, THTPA is ubiquitously expressed with probable post-transcriptional regulation. Much less is known about the recently discovered AThTP. In *E. coli*, AThTP is synthesized by a high molecular weight protein complex from ThDP and ATP or ADP in response to energy stress. A better understanding of these two thiamine derivatives will require the use of transgenic models.

## 1. Introduction

**Figure 1:**
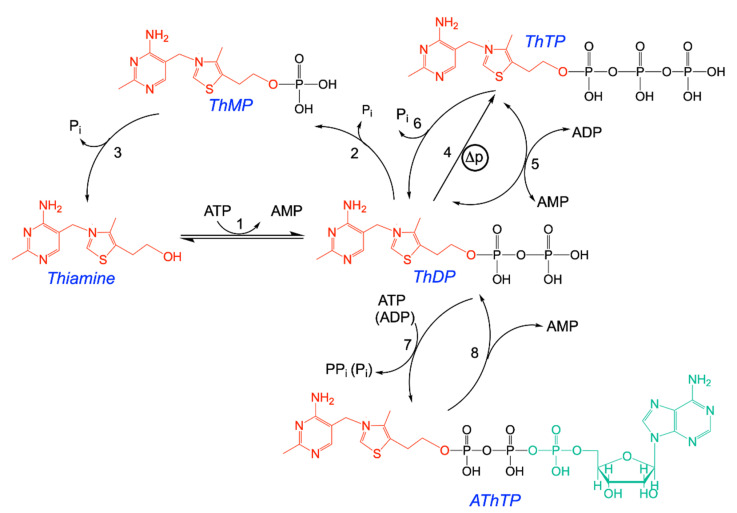
Structural formulas of thiamine and its major derivatives. ThDP is synthesized from thiamine and ATP by thiamine pyrophosphokinase (1). Hydrolysis of ThDP by thiamine pyrophosphatases (2) yields ThMP, which in turn can be hydrolyzed to thiamine by thiamine monophosphatases (3). ThDP can be phosphorylated to ThTP by two mechanisms: mitochondrial ATP synthase (4) and cytosolic adenylate kinase (5). ThTP can be hydrolyzed to ThDP by a very specific cytosolic 25-kDa thiamine triphosphatase (THTPA) but also by other hydrolases (6). ThDP can also be converted to AThTP by a ThDP adenylyl transferase (7). AThTP can be hydrolyzed to ThDP and AMP by a putative AThTP hydrolase (8). Another adenylated thiamine derivative, adenosine thiamine diphosphate (AThDP), not represented here, has been shown to exist in prokaryotes and eukaryotes, but its mechanism of synthesis has not yet been demonstrated in vitro. P_i_, inorganic phosphate; PP_i_, inorganic pyrophosphate; ∆p, proton gradient (adapted from [18]).

Thiamine (vitamin B1) is the precursor for thiamine diphosphate (ThDP), an essential coenzyme for many enzymatic reactions in prokaryotes and eukaryotes [1,2,3] (Figure 1). In animals, the main ThDP-dependent enzymes are the E1 subunits of 2-oxoacid (in particular, pyruvate and oxoglutarate) dehydrogenase complexes and transketolase [4]. These enzymes are essential in oxidative glucose metabolism, explaining why thiamine deficiency leads to severe lesions in particular in tissues with a high oxidative metabolism, such as brain and heart [5]. Thiamine deficiency disorders are still very common, especially in developing countries, and often go unnoticed, resulting in fatal outcome [6,7,8]. It is beyond doubt that the vast majority of the observed symptoms are due to a decreased oxidative metabolism in the nervous system and the heart due to decreased ThDP coenzyme function. Nevertheless, already in the 1950s and 1960s, the idea of a non-coenzyme role of thiamine or some thiamine derivative took shape, probably as a consequence of two observations:

1. Thiamine deficiency leads to selective brain lesions, the diencephalon being the most affected [9,10];
2. Thiamine was reported to be released during electrical nerve stimulation [11].

Considering the now known complexity of the mammalian brain, the first observation might not be so surprising, but it still remains to be explained. Concerning the second observation, a careful review of the literature suggests that many older observations must be taken with care, because of a lack of reliable analytical methods available in these days [12]. Nevertheless, the hypothesis of thiamine non-coenzyme roles continues to be explored in particular in relation to metabolic regulations [13,14]. Indeed, it is now well established that besides ThDP, at least two triphosphorylated thiamine derivatives (Figure 1), thiamine triphosphate (ThTP) and the newly discovered adenosine thiamine triphosphate (AThTP), exist in most prokaryotic and eukaryotic cells [15,16,17], where they might have physiological roles. Moreover, in mammals, ThTP is hydrolyzed by a very specific 25-kDA thiamine triphosphatase (ThTPase, EC 3.6.1.28), coded by the gene *thtpa*. We will refer to this enzyme as THTPA and use the more general term ThTPase for the enzyme activity.

The aim of the present review is to discuss new data obtained on ThTP, AThTP, and their metabolizing enzymatic complexes (Figure 1). 

## 2. Thiamine Triphosphate: Occurrence, Synthesis, Regulation, and Role

### 2.1. Occurrence of ThTP in Living Organisms

The first reports on the existence of ThTP date back to the 1950s in rat liver [19], baker’s yeast [20], rat brain [21], and bacteria [22]. Though the existence of ThTP in these tissues was later confirmed, quantitative aspects should be taken with care as reliable methods for the determination of ThTP became available only in the 1980s with the advent of highly selective and sensitive HPLC methods (for a discussion of this point, see [16,23]). It is also possible that in some cases, ThTP was confounded with AThTP, discovered much later [17]. With state of the art methods, ThTP could be detected, often in highly variable amounts, in practically all animal tissues, plants, fungi, and bacteria [15]. 

In animal tissues, ThTP is constitutionally present in very variable amounts, reflecting tissue-specific balances between synthesis and hydrolysis. In the rat brain, the turnover of ThTP is relatively low with a half-life of 1–2 h [24,25]. In 2010, we published a vast study on the occurrence of ThTP and AThTP in human biopsies [26]. ThTP was found in practically all tissues tested, including all major organs and systems, in the range of less than 1% to over 20% of total thiamine. ThTP levels were generally higher in human compared to the corresponding rodent tissues, which is probably due to the less active 25-kDa ThTPase, an important regulator of ThTP levels in animals (see below). While in the brain, ThTP is mainly localized in mitochondria and mitochondria-rich fractions (synaptosomes), in skeletal muscle and liver, it is mainly in the cytosolic fraction, most probably reflecting different mechanisms of synthesis (see Section 2.2) [25,26,27].

On the other hand, in *E. coli,* it has to be induced by a carbon source in amino acid-deficient media and can rapidly reach up to 50% of total thiamine [28,29]. As ThTP synthesis in *E. coli* only occurs in a medium devoid of amino acids, it was suggested that ThTP might be involved in a response to amino acid starvation [28].

In plants, ThTP is found mainly in the plastids and mitochondria of the shoots rather than the roots and peaks early during the light period and the authors suggest that it may signal ThDP sufficiency [29].

### 2.2. Two Different Mechanisms of Synthesis of ThTP: Adenylate Kinase and ATP Synthase

Different mechanisms were put forth concerning the synthesis of ThTP, among which the most straightforward is the transfer of a phosphate group from ATP to ThDP according to the reaction ThDP + ATP ⇆ ThTP + ADP catalyzed by a ThDP:ATP phosphotransferase [30] (for a review, see [16]). However, such a mechanism was never confirmed.

It is important to emphasize that ThTP, like ATP and other nucleoside triphosphates, is an “energy-rich” compound with two phosphoanhydride bonds and a high phosphoryl transfer potential. Its synthesis thus requires a significant energy input.

It has now been firmly established that ThTP may be formed by two different mechanisms. The first mechanism is the synthesis of ThTP by cytosolic adenylate kinase 1 (AK1, myokinase, EC 2.7.4.3) according to the reaction first reported by [28]: ThDP + ADP ⇆ ThTP + AMP instead of the more canonical reaction 2 ADP ⇆ ATP + AMP. Hence, in mammalian liver and skeletal muscles, ThTP is essentially synthesized by cytosolic AK1, explaining why ThTP is mainly found in the cytosolic fraction in these tissues [26,31,32,33].

Bacterial AKs (for instance, *E. coli* AK shares several regions of high homology with AK1 [34]) are also able to synthesize ThTP according to the above reaction [35]. Hence, ThTP synthesis seems to be a rather general property of type 1 AKs. With recombinant enzymes, ThTP synthesis is over 10^6^ times lower than ATP synthesis [35], but this might be enough to ensure a constitutional and cytosolic synthesis of ThTP. However, ThTP synthesis by AK1 is not the only mechanism as ThTP levels are hardly decreased in tissues of transgenic AK1 knockout mice [36].

The second mechanism of ThTP synthesis seems to be related to ATP synthase (EC 7.1.2.2, F_o_F_1_-ATPase). Indeed, energized isolated rat brain mitochondria are able to synthesize ThTP from ThDP and P_i_, which can then be exported in exchange with P_i_ or nucleotides [32]. This synthesis is tightly coupled to the respiratory chain and depends on the existence of a transmembrane H^+^ gradient, suggesting that ThTP is synthesized by a mechanism similar to ATP and involving ATP synthase. Interestingly, only brain but not liver mitochondria were able to synthesize ThTP. This suggests a cell-specific regulation of ThTP by mitochondria. In *E. coli*, ThTP can accumulate to over 50% of total thiamine and is most probably synthesized by ATP synthase [37]. Very recently it was shown that in *Arabidopsis* shoots, ThTP synthesis oscillates during the diel period and may accumulate during the early light period [29]. This synthesis occurs in the plastids and mitochondria, is dependent on the proton gradient, and is coupled to photosynthetic processes, again suggesting the involvement of chloroplast and mitochondrial ATP synthases. ThTP synthesis has not yet been reported with purified and reconstituted ATP synthase, but the purified F_1_ fragment may hydrolyze ThTP at a very low rate [37].

### 2.3. Regulation of Intracellular ThTP Concentrations

Mitochondria, *E. coli* and chloroplasts, are able to synthesize ThTP, strongly linking this synthesis to proton gradient-dependent energy processes. It is not established whether this synthesis is a mere side reaction of ATP synthase or a physiologically relevant process. The fact that mitochondrial ThTP synthesis is tissue specific (brain mitochondria may synthesize ThTP, while liver mitochondria may not [32]) would suggest the latter. With this respect, it is also interesting to note that ThTP synthesis seems to be decreased in cardiovascular system biopsies of patients with heart failure syndrome [26]. Indeed, the ratio ([ThTP + [ThMP])/([Thiamine] + [ThMP]), which we called the “thiamine phosphorylation ratio”, was low (<1) in these patients, while it did not correlate with age or underlying neoplastic disease. As these patients suffer from metabolic shifts and decreased energy generating processes, this observation emphasizes the coupling of ThTP synthesis to cell energy metabolism. Indeed, ThTP steady-state concentrations are a balance between synthesis and hydrolysis [24,25]. In case of energy shortage, hydrolysis would prevail, leading to accumulation of ThMP (Figure 1). Intracellular ThDP concentrations are one or two orders of magnitude higher than those of ThTP and ThMP, so that no significant changes in ThDP levels are observed when ThTP is hydrolyzed but ThMP accumulates.

An intriguing observation was that some tissues have an extremely high proportion of ThTP compared to total thiamine, sometimes exceeding 70%. This is the case for pig [33] and chicken [38] skeletal muscles and fish electric organs (which are ontogenetically derived from skeletal muscle) from *Torpedo marmorata* [39] and *Electrophorus electricus* [40], all tissues with high AK1 activity. Why these tissues specifically accumulate ThTP remained a mystery until it was understood that, though AK1 is responsible for ThTP synthesis in the tissues (but also in the skeletal muscle of other species), THTPA hydrolyzes ThTP in a highly efficient manner except in fish, birds, and pigs (Table 1). Indeed, fish have a specific THTPA, but its activity is orders of magnitude lower than that of the mammalian enzymes and no significant cytosolic ThTPase activity has ever been measured in fish tissue [15,41]. Birds have completely lost a region encompassing 30 genes including the gene coding for THTPA [41]. Due to several amino acid changes, the domestic pig expresses a catalytically inactive THTPA [42]. Hence, the accumulation of ThTP in skeletal muscle and electric organs is the consequence of high AK 1 activity combined with low or absent THTPA activity. These results emphasize the importance of THTPA in the regulation of cytosolic ThTP concentrations.

Rats, genetically selected for high-alcohol sensitivity, have higher ThTP levels in liver and brain compared with low-alcohol sensitivity animals [43], but the significance of this observation remains to be established.

Based on expression analysis, a recent study suggested a correlation between the pituitary tumor-transforming gene family and ThTP metabolism [44], but obviously they confounded ThTP with thymidine triphosphate.

### 2.4. The Special Case of ThTP Synthesis in E. coli

For a long time, we have been looking for conditions that would alter intracellular ThTP concentrations in a simple system, such as cultured neuroblastoma cells; however, no reproducible conditions were found (unpublished results). We now know that in these cells, ThTP is synthesized constitutionally by AK and that THTPA is not expressed at the protein level in neuroblastoma cells in its active from, though the mRNA is present and ATP synthase does not play a significant role in ThTP synthesis in these cells [26].

Therefore, we looked for a simpler model, such as *E. coli.* Indeed, we found that in *E. coli* cells, ThTP is detected under very specific conditions: aerobic growth, amino acid starvation, and a suitable sugar or sugar-derived carbon-source [28,37], conditions recently confirmed by another group [29]. ThTP synthesis is inhibited by amino acids, with some (Cys, Glu, Arg, and Trp) being particularly effective, pointing to a metabolic regulation of ThTP synthesis. The requirement of a carbon source (different from amino acids) is explained by the necessity of generating a proton gradient. However, not all carbon sources that generate a proton gradient were equally efficient to significantly boost ThTP synthesis. Indeed, the most efficient were those feeding into glycolysis (d-glucose, d-fructose, d-gluconate, pyruvate, and l-lactate), while intermediates of the citric acid cycle (citrate, oxoglutarate, fumarate, malate, and oxaloacetate) were inefficient. Some (acetate, glutamate, succinate) were of intermediate efficiency.

**Figure 2:**
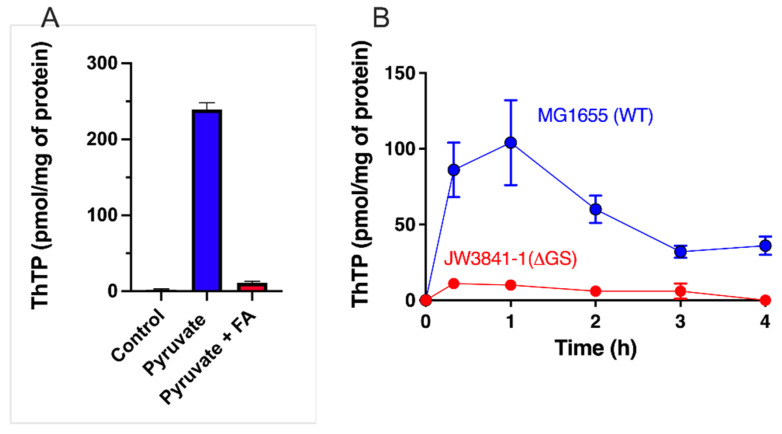
Importance of the pyruvate–glutamine axis for ThTP synthesis in *E. coli*. (**A**) Inhibition of ThTP synthesis by fluoroacetate (FA). *E. coli* cells from the BL21 strain were grown in LB medium and then transferred into a M9 minimal medium (devoid of amino acids) for 20 min (37 °C, 250 rpm) in the absence or the presence of either pyruvate (10 mM) or pyruvate (10 mM) + fluoroacetate (10 mM) and ThTP was determined by HPLC [28]. (**B**) ThTP synthesis is strongly decreased in a strain specifically deficient in glutamine synthetase (JW3841-1 derived from parent strain MG1655/K12 - ATCC 47076). Both strains were grown in minimal medium in the presence of 10 mM D-glucose for the indicated times and ThTP was determined as above. Single gene deleted strain JW3841-1 [46] (CGSC # 10775) was obtained from the Genetic Resource Center (Yale University, New Haven, CT, USA) (data are from [47] and expressed as mean ± SD).

**Figure 3:**
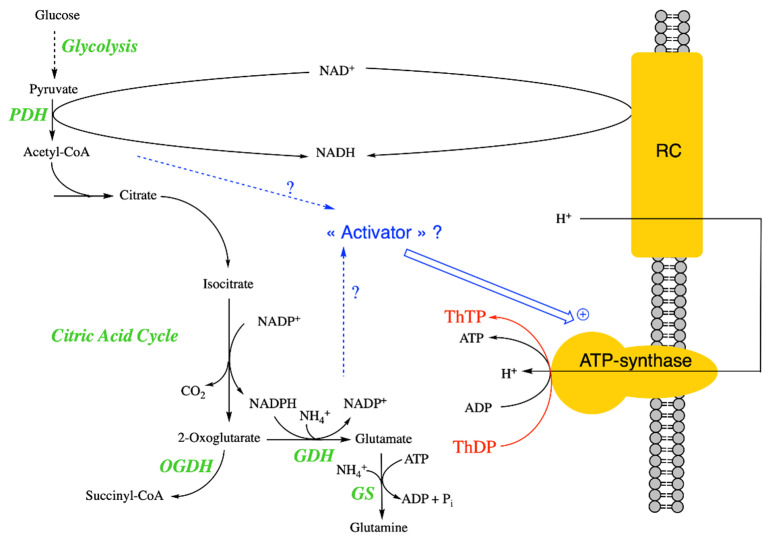
Mechanism of ThTP synthesis in *E. coli* and possible regulation. It seems that ThTP synthesis by a chemiosmotic process requires an activator synthesized either during the early reactions of the Krebs cycle or after the branching point towards glutamate and glutamine. ThTP synthesis by AK is negligible in *E. coli* under these conditions. Note that *E. coli* isocitrate dehydrogenase requires NADP^+^ and not NAD^+^ as co-substrate [48] (PDH, pyruvate dehydrogenase; GDH, glutamate dehydrogenase; GS, glutamine synthetase).

This suggests that pyruvate oxidation is an essential condition for ThTP synthesis [37]. When pyruvate oxidation is blocked by fluoroacetate, which is transformed into fluorocitric acid, an inhibitor of aconitase [45], no ThTP synthesis occurs (Figure 2A). Pyruvate is required because it yields NADH, a substrate for the respiratory chain. The requirement for oxygen was later recognized when it was found that ThTP is synthesized by ATP synthase, requiring an active respiratory chain [37]. However, it appears that pyruvate is also required because it yields an “activator” essential for ThTP synthesis. This activator is supposedly synthesized either during the early reactions of the Krebs cycle or after the branching point towards glutamate and glutamine (Figure 3). In order to test this hypothesis, we used an *E. coli* strain deleted in glutamine synthetase (Figure 2B). This strain was unable to accumulate ThDP, suggesting that glutamine, or a downstream metabolite, might activate ThTP synthesis or the accumulation of a metabolite upstream of glutamine would inhibit ThTP synthesis.

These results again underscore the importance of a metabolic regulation of ThTP synthesis in *E. coli* and possibly the existence of a regulatory switch located between pyruvate and oxoglutarate [37] (Figure 3).

### 2.5. Possible Physiological Roles of ThTP in Nerve Tissue

#### 2.5.1. A Specific Role of ThTP in Nerve Excitability

Historically, it was thought that ThTP might have a specific role in membrane excitability and nerve conduction. This hypothesis was, among others, based on the following two observations:

1. The existence of very high levels of ThTP in electric organs, rich in Na^+^ channels [39,40], but this is probably just a coincidence due to high AK1 and low THTPA activity in these tissues as mentioned above.
2. The electrical stimulation of isolated nerves leads to a release of thiamine, probably resulting from a dephosphorylation shift of higher thiamine phosphoester, such as ThDP and ThTP [11].

In agreement with the latter observation, we could show that intermittent light stimulation of the photosensitive baboon *Papio papio* resulted in a decreased [ThTP]/[ThMP] ratio of the occipital (visual) cortex in the animals [49]. In view of what we now know about the synthesis of ThTP in nervous tissue, these results might just reflect a decreased synthesis of ThTP by ATP synthase under highly energy consuming conditions (see Section 2.3). Under such conditions, hydrolysis of ThTP would continue, leading to decreased ThTP and increased ThMP levels. ATP shortage might also lead to decreased synthesis of ThDP by thiamine di(pyro)phospkokinase (TPK1) according to the reaction thiamine + ATP ⇆ ThDP + AMP (EC 2.7.6.2), the only known enzyme involved in ThDP synthesis in animals.

Hence, there is no strong evidence that ThTP may play a role in nerve excitability.

#### 2.5.2. ThTP and Neurotransmitter Release

Several studies suggest an involvement of ThTP in neurotransmitter release. Hence, ThTP (>0.1 mM), but also ThDP, induced a Ca^2+^-dependent dopamine release in the rat striatum [50]. This effect might be mediated by extracellular purinoceptors. An ancient study suggested a role of thiamine, and possibly ThTP, in cholinergic transmission in the electric organ of *Torpedo marmorata* [39]. This was based on the presence of very high concentrations of thiamine and its phosphate esters in the cytoplasm of the electric organ.

#### 2.5.3. ThTP and Protein Phosphorylation

As pointed out above, ThTP, like ATP, has a high phosphate group transfer potential and could thus be a phosphate donor in protein phosphorylation reactions. This hypothesis was tested using [γ-^32^P]-ThTP on membranes of the acetylcholine-rich electric organ of *T. marmorata*. In these preparations, ThTP indeed phosphorylated the protein rapsyn, associated with the nicotinic acetylcholine receptor and essential for the clustering of the receptors at the neuromuscular junction [51]. This phosphorylation occurred at low ThTP concentrations (5–25 µM) on histidine residues in the absence of added kinases. The physiological relevance of this phosphorylation, however, remains to be attested.

#### 2.5.4. ThTP and Membrane Chloride Permeability

ThTP was also thought to be directly involved in the regulation of ion channels in nerve cells [52]. When synaptoneurosomal membrane vesicles prepared from rat brain were incubated with thiamine or ThDP in order to increase intravesicular ThTP levels, the ThTP levels correlated with chloride membrane permeability [53,54,55]. In order to identify possible chloride channels involved in these permeability changes, we tested the addition of ThTP to excised inside-out patches in cultured mouse neuroblastoma cells. Under these conditions, ThTP at relatively low concentrations (<10 µM) activated very high conductance chloride channels of 300–400 pS, the so-called maxi chloride channel [56]. At this time, neither the identity nor the role of this channel was known. It now appears that its core component is the organic anion transporter SLCO2A1, which, in its resting (closed) state, functions as a prostaglandin transporter and in its active (open) state as a channel releasing mainly chloride and ATP [57]. This channel is thought to be involved in cell volume regulation and purinergic cell-to-cell signaling.

#### 2.5.5. ThTP and Glutamate Dehydrogenase

ThTP (1 and 10 µM) allosterically activates commercial preparations of the purified glutamate dehydrogenase (GDH) from bovine liver [14], though no in vivo data are available. GDH is at the crossroads of carbon and nitrogen metabolism. Indeed, it is thought that in mammals, the reaction catalyzed by GDH is favored in the catabolic sense (synthesis of 2-oxoglutarate from glutamate). 2-Oxoglutarate can be either catabolically decarboxylated by 2-oxoglutarate dehydrogenase and continue through the citric cycle or anabolically funneled into amino acid metabolism by transamination. In microorganisms and possibly plants, GDH functions in the anabolic sense of glutamate synthesis from 2-oxoglutarate and NH_4_^+^ and may play a role in nitrogen assimilation [58]. Hence, the balance of these pathways is essential for the energy status of the cells in all organisms.

## 3. Thiamine Triphosphatases

No specific ThTPases have been identified in microorganisms, fungi, or plants, but a membrane-associated and a cytosolic ThTPase have been extensively studied in animal cells. In addition to these two ThTPases, myosin is able to hydrolyze ThTP to ThDP [59]. ThTP may bind to a regulator site on myosin, but it does not seem to affect muscle contraction [60].

*E. coli* represents a particularly interesting case. In *E. coli*, ThTP accumulation is always transitional, with a maximum after 1 or 2 h. Thereafter, ThTP levels steadily decrease (Figure 2B, [28]). Decreased ThTP levels are concomitant with increased ThDP levels, suggesting a mechanism of hydrolysis that starts to prevail over synthesis. No specific enzymatic mechanism of ThTP hydrolysis has been evidenced so far in *E. coli*. However, ThTP is hydrolyzed by the purified F_1_ subunit of *E. coli* ATP synthase with an apparent K_m_ of 40 µM but a very low k_cat_ of 1.5 min^−1^ [37]. This result is not surprising if ATP synthase is responsible for ThTP synthesis, and one can expect that, as for ATP, ThTP is hydrolyzed by F_1_-hydrolyzing activity.

### 3.1. Mammalian Membrane-Associated ThTPase

A membrane-associated ThTPase is present ubiquitously in mammalian cells. It has not yet been identified at the molecular levels and it is not clear whether this enzymatic activity results from one or several enzymes and to what level it is physiologically relevant. This enzyme retained some attention as thiamine was thought to have a role in nerve excitability, a hypothesis now more or less abandoned in favor of a more metabolic role [16,23].

This ThTPase activity was first reported in the rat brain [61], but it seems to exist in most mammalian tissues, where it is found in all particulate fractions. It is activated by both Mg^2+^ and Ca^2+^ and has a K_m_ in the millimolar range for ThTP. It is competitively inhibited by low concentrations of ATP and ADP, with IC_50_ values of 20 µM for ATP and 75 µM for ADP [62]. These results suggest that the active site has a much higher affinity for adenine nucleotides than for thiamine compounds. It remains possible that the enzyme would be a nucleoside triphosphatase with broad substrate specificity. 

A membrane-associated ThTPase with somewhat different properties was later described in electric organs [40,63,64] and skeletal muscle [65]. It is not clear whether it is distinct from the brain enzyme or just an isoform with different properties. The distinctive feature of this ThTPase is its strong activation by chaotropic anions (I^−^ > SCN^−^ > NO_3_^−^ > Br^−^ > Cl^−^), a property not observed for the brain enzyme, which, on the contrary, is inhibited by anions. Though the enzyme could be solubilized by detergents, the activity was lost during purification [66].

Very recently, the solubilization and the partial purification of the chicken liver enzyme was reported [67]. The ThTPase activity was coeluted with ATP and ITP hydrolyzing activities in a high molecular mass fraction and the K_m_ for ThTP was about 2 mM.

From what we presently know, it is likely that the membrane-associated ThTPase activities are not from genuine ThTPases but rather nucleoside triphosphatases with broad substrate specificity.

A membrane-associated brain synaptosomal protein, binding thiamine phosphorylated derivates, including ThTP, seems to have ThTPase activity [68,69]. The authors report a pH optimum of 7.4 and an apparent K_m_ of 52 µM, which is somewhat lower than reported earlier for the membrane-associated ThTPase. It is not clear whether this protein is identical to the membrane-associated brain ThTPase mentioned above.

### 3.2. Soluble 25-kDa ThTPase (THTPA)

#### 3.2.1. Characterization, Structure, and Evolution of THTPA 

A soluble ThTPase (THTPA) was first described in 1972 [70]. This enzyme proved to be more interesting than the membrane-associated enzyme. Though mainly cytosolic, one study suggested that a soluble ThTPase with somewhat different properties exists in the mitochondrial matrix and intermembrane space [71]. It is not clear whether it is the same or a different enzyme.

**Figure 4:**
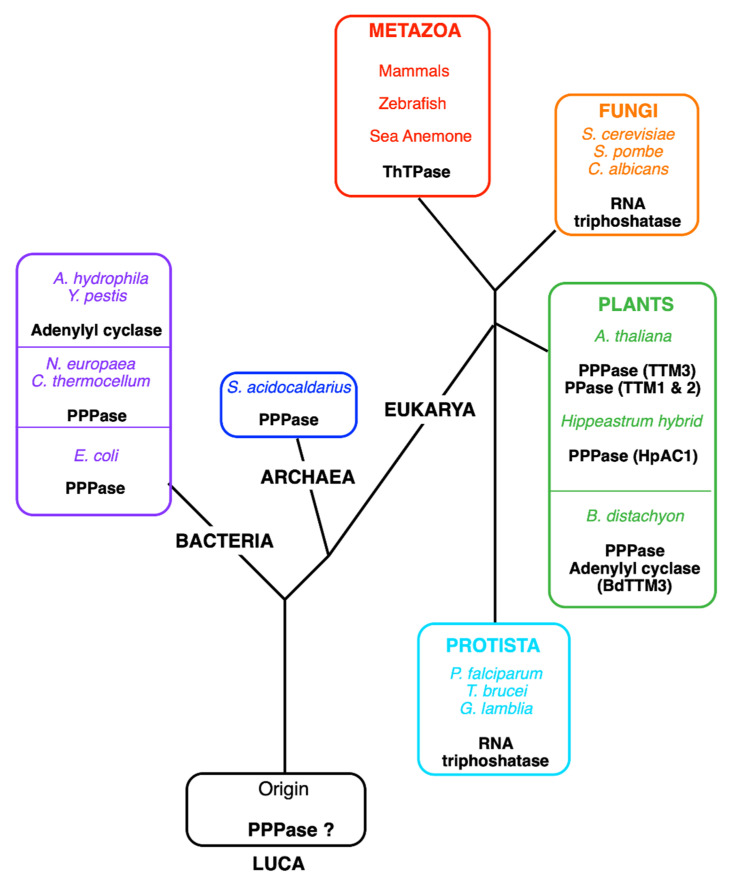
Tentative phylogenetic tree of the TTM (CYTH) superfamily. Currently, five different enzyme activities have been identified for members of the CYTH superfamily: tripolyphosphatase in bacteria (*C. thermocellum*, *N. europaea*, *E. coli*), an Archaea (*Sulfolobus acidocaldarius)* and plants (*A. thaliana* and *Hippeastrum* hybrids), adenylyl cyclase (in *A. hydrophila* and *Y. pestis*), RNA triphosphatase (in fungi and some protozoans), and ThTPase (in most metazoans, including vertebrates except for birds). Lately, pyrophosphatase activity (PPase) was recognized in *A. thaliana* TTM1 and TTM2, proteins containing a TTM and a uridine kinase domain [87]. The plant *B. distachyon* (BdTTM3) shares high triphosphatase activity and low adenylyl cyclase activity [84]. We hypothesize that the original activity in the Last Universal Common Ancestor (LUCA) was the hydrolysis of low molecular mass polyphosphates.

THTPA was purified to homogeneity by A. Makarchikov in 1992 from bovine brain [72]. This enzyme requires Mg^2+^ as cofactor, but it is inhibited by Ca^2+^, but its most interesting property is its near absolute specificity for ThTP as substrate and a relatively low K_m_ (43 µM), suggesting that it is a genuine ThTPase. The recombinant human ThTPase was cloned, produced in *E. coli*, purified, and sequenced in 2002 [73]. The human enzyme is composed of 230 amino acids, has a predicted molecular mass of 25.55 kDa, and is localized to chromosome 14q11.2. ThTPase activity and THTPA mRNA are widely distributed in mammalian tissues, with no apparent specificity for nervous tissue [73,74]. Shortly after the publication of the primary structure of THTPA, Iyer and Aravind detected the presence of a common motive between mammalian THTPA and the CyaB protein from *Aeromonas hydrophila*, a non-canonical adenylate cyclase [75]. More importantly, they showed that “the CyaB like adenylyl cyclase and the mammalian ThTPases define a novel superfamily of catalytic domains called the CYTH (contraction of CyaB and THTPA) domain that is present in all three superkingdoms of life”. The conserved CYTH motive is mainly formed by the sequence EXEXK (where X is any amino acid) characteristic of this family and is an ancient enzymatic domain, present in the Last Universal Common Ancestor (LUCA) and involved in nucleotide or organic phosphate metabolism [75]. In 2006, Gong et al. noticed similarities between the crystal structure of Cet1 RNA triphosphatase (EC 3.1.3.33) from *Saccharomyces cerevisiae* and the structures of bacterial and archaeal CYTH proteins [76]. All these enzymes had an active site composed of an eight-strand ß-barrel forming a topologically closed tunnel. They proposed that this tunnel fold was the prototype of a larger superfamily termed triphosphate tunnel metalloenzymes (TTMs), including the CYTH branch and able to bind triphosphorylated substrates, such as ATP and ThTP, and requiring divalent metal ions as activators. Some bacterial members of this family, such as the *N. europaea* (NeuTTM) and *E. coli* (YgiF), are functional inorganic tripolyphosphatases [77,78] and so are the *A. thaliana* ortholog AtTTM3 [79] and the *Hippeastrum* HpAC1 protein [80] (Figure 4). The latter was considered an adenylate cyclase until its enzymatic and structural characterization revealed predominant triphosphatase activity. Hence, the authors suggested renaming the protein HpPP1 or HpTTM1 [80]. In *A. thaliana*, two other TTMs, TTM1 and TTM2, are involved respectively in pathogen resistance and leaf senescence [81]. Surprisingly, both have a higher activity towards PPi than towards triphosphorylated substrates and both carry a uridine kinase domain in addition to the CYTH domain. The only archaeal TTM protein characterized enzymatically proved to be a tripolyphosphatase [82].

The picture that emerges is that TTM proteins evolved from an inorganic tripolyphosphatase acquiring new substrate specificities in fungi and some protists (RNA triphosphatase) and animals (ThTPase) or even new catalytic mechanisms in some bacteria (CyaB like adenylyl cyclase) [83]. The recombinant form of the plant *Brachypodium distachyon* (BdTTM3) even shares high triphosphatase activity (binding both tripolyphosphate and ATP) and low adenylate cyclase activity and might be involved in the mechanism underlying responses to wounding stress [84].

Interestingly, all metazoans (from sea anemone to humans) possess one TTM protein with ThTPase activity. Birds are a notable exception: a whole region of 30 genes, including the *thtpa* gene, has disappeared on chromosome 14 [41], leaving this branch as the only one known without a TTM protein. Moreover, fish and pigs, though retaining their THTPA orthologs, have practically no activity [15,41,42], which is probably the reason why ThTP accumulates in these tissues (see Section 2.3 and Table 1).

**Figure 5:**
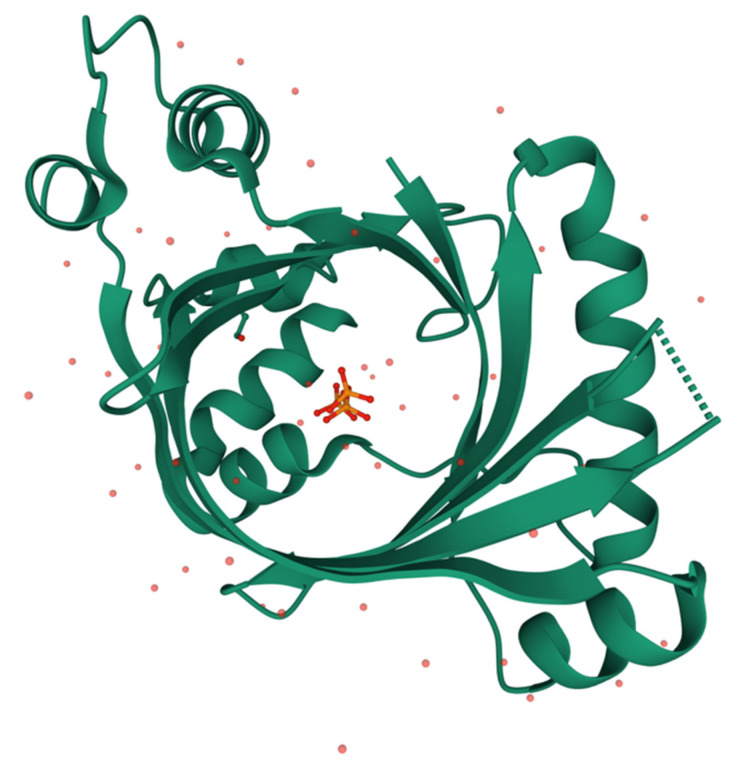
Three-dimensional structure of human THTPA in complex with inorganic tripolyphosphate (RCSB PDB ID 3TVL, https://www.rcsb.org) [41]. Image created using Mol* [88].

The mammalian enzyme is well conserved, with an estimated evolutionary period of 4.4 million years, corresponding to an average rate of amino acid substitutions of 1.41 × 10^−9^ per site per year [85]. The three-dimensional structure of mammalian THTPA was obtained by NMR [86] and crystallography [41], confirming an open tunnel structure of the active site, closed by substrate binding (Figure 5). Mass spectrometry and site-directed mutagenesis confirmed a high affinity (micromolar range) and high specificity for ThTP, probably due to the Trp-53 residue responsible for a stacking interaction with the thiazole ring of ThTP [41]. On the other hand, Tyr-39 and Lys-65 are important residues for catalysis forming a catalytic dyad [41].

Though, Mg^2+^ is required for catalytic activity, and Mg^2+^ tightly binds to ThTP, it seems that Mg^2+^ dissociates from ThTP and binds to a different site, probably including the two glutamate residues of the EXEXK CYTH motive [41].

#### 3.2.2. Possible Physiological Roles of THTPA and Regulation

From the data mentioned above, the very high cytosolic concentrations of ThTP in animal tissues where THTPA is either absent (chicken skeletal muscle) or poorly active (pig skeletal muscle, *E. electricus* electric organ) suggest that THTPA is indeed involved in the regulation of cytosolic ThTP concentrations. This is further emphasized by its high specificity for ThTP as substrate. However, the physiological significance of this reaction remains obscure.

THTPA is mainly expressed in adult tissues. In the rodent brain, using in situ hybridization and immunohistochemistry, the strongest staining was observed with both methods in hippocampal pyramidal neurons, as well as cerebellar granule cells and Purkinje cells [89]. Soluble ThTPase activity is low in cultured and embryonic cells [26], but as mRNA is abundant, we proposed that the expression of the protein may be controlled post-transcriptionally or by post-translational modification, possibly through the highly conserved 200-nucleotide 3′-untranslated mRNA region [74]. With this respect, it should be emphasized that *thtpa* expression may be controlled by genes (Ndrg-1, melanotransferrin) involved in cancer cell models [90,91].

A recent study suggested that THTPA, among several other proteins, might serve as biomarkers for stratification of tumor subtypes in breast cancer [92].

We produced transgenic mice overexpressing bovine ThTPase in mice with the aim of making the animals devoid of ThTP. These mice indeed had about two-fold increased ThTPase activities, but ThTP levels were unchanged [16]. Indeed, mouse ThTPA is catalytically highly efficient and ThTP levels are already very low. Moreover, we did not know at the time that, except for skeletal muscle, most of the ThTP is produced intramitochondrially by ATP synthase. These mice did not display any evident phenotypic specificities. 

Another possibility would be that cytosolic ThTP accumulation would be cytotoxic. This is, however, clearly not the case in pig and chicken skeletal muscle and electric organs. 

Linster et al. suggested the interesting hypothesis that THTPA is involved in metabolite repair, i.e., the regeneration of the coenzyme ThDP from ThTP, formed as a byproduct of other reactions, such as adenylate kinase or ATP synthase [93]. However, even when THTPA activity is absent, ThTP, except for pig and chicken skeletal muscle and electric organs, remains low compared to ThDP [15,26,33,40].

**Table 1:** Relative ThTP content and AK1 and THTPA activities in various animal species. This table shows a correlation between, on the one hand, ThTP levels and, on the other hand, low ThTPA activity and high AK1 activity.

Species	AK1 (µmol min^−1^ mg^−1^ Protein)	THTPA (nmol min^−1^ g^−1^ ww)	ThTP (nmol g^−1^ ww)
**Fish** *E. electricus* electric organ *E. electricus* brain *T. marmorata* electric organ	— — —	n. d. ^1^ n. d. ^1^ n. d. ^1^	3.9 ± 0.5 (87%) ^1^ 0.37 ± 0.05 (7.1%) ^1^ 45 ± 4 (38%) ^2^
**Birds** Chicken skeletal muscle Chicken brain	1.6 ± 0.1 ^3^ —	n. d. n. d.	3.7 (71) ^4^ 0.92 (8.4%) ^4^
**Mammals** Pig skeletal muscle Mouse skeletal muscle (wt) Mouse skeletal muscle (AK^−/−^) Mouse brain (wt) Mouse brain (AK^−/−^)	1.03 + 0.15 ^3^ 1.3 ± 0.2 ^3^ — 0.16 ± 0.2 ^3^ 0.02 ± 0.01 ^3^	12.5 ± 0.5 ^4,6^ 42 ^3^ — 280 ± 30 ^5^ —	20 (64%) ^4^ 0.026 ± 0.006 (0.35%) ^3^ 0.026 ± 0.008 (0.36%) ^3^ 0.009 ± 0.003 (0.09%) ^3^ 0.014 ± 0.01 (0.14%) ^3^

^1^ [40]; ^2^ [39]; ^3^ [36]; ^4^ [15]; ^5^ [74]; ^6^ This activity is probably the result of other ThTPases than THTPA. (—, not measured; n. d.; not detected; ww, wet weight).

We tried to produce *thtpa* knockout mice [94]. KO mice were generated from embryonic stem cell clones obtained from the Knockout Mouse Project (KOMP) repository at UC Davis (www.komp.org). We could generate chimera derived from the stem cells and all were partly THTPA^+/+^ and partly THTPA^+/−^. However, none of the chimerae transmitted the mutation: 12 chimerae were generated and a total of 455 pups were obtained. We genotyped all of them and, surprisingly, they were all wild type. This could mean either that *thtpa* is essential for the differentiation of functional spermatozoids or the introduction of the KOMP cassette led to the disruption of genes essential for spermatozoid differentiation. A role of *thtpa* in sperm cells is supported by a very high expression of its mRNA in testis with the highest expression observed in poorly differentiated spermatogonia close to the basal lamina, while no signal was observed in spermatozoids [74].

## 4. Adenylated Thiamine Nucleotides

### 4.1. Discovery, Chemical Synthesis, and Chemical Properties

When working on the synthesis of ThTP in *E. coli* cells, we observed a new chromatographic peak appearing under conditions of carbon starvation [28], hence essentially conditions opposite to those inducing the appearance of ThTP. Here, it must be emphasized that analysis of thiamine compounds relies on the precolumn derivatization to fluorescent thiochrome derivatives [95]. This new fluorescent peak was not observed in the absence of thiamine-specific precolumn derivatization and had fluorescence excitation and emission spectra compatible with a thiochrome derivative [96], suggesting that it might correspond to a new thiamine derivative. After purification and identification using a combination of mass spectrometry and NMR, it proved to be an adenylated thiamine derivative, which could be either considered as thiaminylated ATP or adenylated ThDP (Figure 1). While many adenylated B vitamins are known (i.e., NAD^+^, FAD, acetyl-CoA) and are considered to be remnants from an RNA world [97], this was the first adenylated thiamine compound ever to be identified. As this molecule exists in only very small concentrations (micromolar and submicromolar concentration range) as is the case for most vitamins as well as its opposite regulation in *E. coli* compared with ThTP, we preferred the term adenosine thiamine triphosphate (AThTP) rather than thiaminylated ATP, so as to emphasize its role as a vitamin derivative. AThTP was shown to be present not only in *E. coli* (where it may account for up to 15% of total thiamine), but also in much smaller concentrations in animals, fungi, and the roots of *A. thaliana* plants [17,96,98]. The latter observation could however not be reproduced in another study [29].

Despite a relatively broad distribution, nothing is known about the regulation of the synthesis or hydrolysis of ThTP in eukaryotic cells and it cannot be excluded that AThTP is a waste product.

AThTP could be chemically synthesized by condensation of ADP and ThDP catalyzed by N,N′-dicyclohexylcarbodiimide [17,96] and identified by a combination of ESI tandem MS and ^1^H- ^13^C- ^31^P-NMR. More recently, improved methods of synthesis have been proposed [99,100].

During this synthesis, we observed the formation of another compound, which proved to be adenosine thiamine diphosphate (AThDP). This compound, could be detected in small amounts in *E. coli* and mammalian liver, suggesting that it is a naturally occurring molecule [96].

Based on ^1^H-NMR and molecular modeling, we proposed that both AThTP and AThDP exist under a U-shaped folding, bringing together the adenine and thiamine moieties [96]. In such structures, the C-2 proton of the thiazolium ring of thiamine, which is required for the catalytic activity of ThDP, is embedded in a hydrophobic environment formed by the adenine and aminopyrimidine rings, making it improbable that AThTP and AThDP are catalytically active.

### 4.2. Regulation of AThTP Synthesis in E. coli

As mentioned above, AThTP is synthesized in *E. coli* under conditions of carbon starvation, but a closer analysis revealed a much more complex situation [35].

1. It is not the absence of a carbon source *per se* that triggers AThTP synthesis, but the absence of its metabolization. Hence, AThTP accumulates in the presence of glucose when glycolysis is inhibited by iodoacetate, or in the presence of lactate when the respiratory chain is inhibited by KCN.
2. AThTP synthesis probably requires a low molecular weight factor seemingly synthesized from pyruvate. Indeed, uncouplers, such as CCCP, induce ThTP synthesis only in the presence of pyruvate or a pyruvate-yielding substrate (D-glucose or L-lactate).
3. ThTP is an inhibitor of AThTP synthesis, explaining at least in part that both compounds never accumulate together.

Hence, AThTP may accumulate under two different conditions of severe energy stress: absence of an energy substrate (or inhibition of its metabolization) and uncoupled pyruvate oxidation. However, there is no obvious link with the stringent response to carbon starvation or catabolite repression [35].

### 4.3. AThTP Is Synthesized by a High Molecular Weight Complex in E. coli

An AThTP synthesizing activity, according to the reaction ThDP + ATP (ADP)⇌ AThTP + PP_i_ (P_i_), was isolated from an *E. coli* supernatant after precipitation and Sephadex G-200 chromatography [101]. The enzyme activity was eluted in two nearly equal peaks of 355 and 190 kDa, the first probably a dimer of the second. This finding was confirmed more recently and both peaks contained multiple protein bands when analyzed by SDS-PAGE [102].

The synthesis required ThDP and either ATP or ADP as substrates. A divalent cation, either Mn^2+^ or, the more physiological, Mg^2+^ was also required. The kinetics were complex, with a lag period and a sigmoidal behavior with respect to ADP (Hill coefficient = 2.1), in agreement with a multisubunit complex. Further purification systematically led to the loss of activity, possibly because of the dissociation of the complex. We tentatively called this enzyme complex a ThDP adenylyl transferase [101].

Another important point is that the enzyme is activated by a low molecular weight organic compound present in bacterial extracts [101]. This would be in agreement with the requirement of a metabolite of pyruvate oxidation in vivo (see Section 4.2). 

### 4.4. AThTP Hydrolysis in E. coli

**Figure 6:**
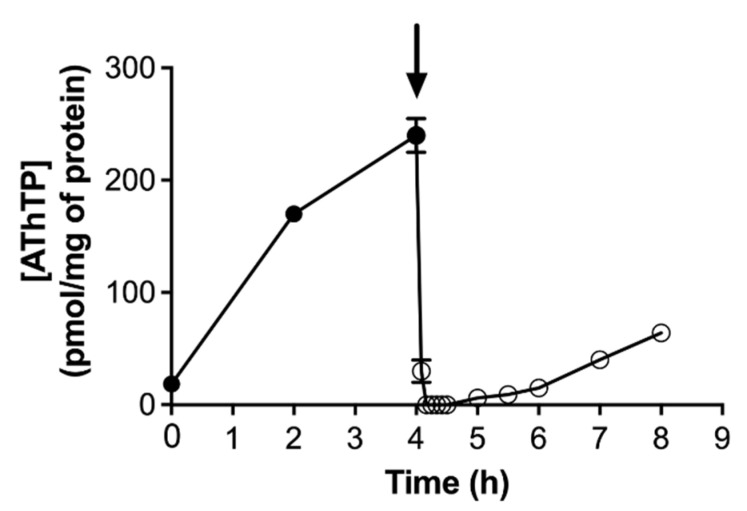
AThTP levels as a function of time in *E. coli* BL21 cells transferred to a minimal medium and incubated at 37 °C at 250 rpm in the absence of a carbon source. Aliquots were taken for determination of thiamine derivatives. The arrow indicates the addition of 10 mM D-glucose (mean ± SD, *n* = 3) (adapted from [103]).

In contrast to ThTP accumulation, which is transient, AThTP accumulation is not: its levels remain high as long as the stress condition is present. However, addition of a carbon source to *E. coli* cells under carbon starvation will lead to the immediate disappearance of AThTP (Figure 6). The rapidity with which this disappearance occurs suggests the activation of an enzymatic mechanism of hydrolysis. An enzyme present in the bacterial membrane extracts is able to hydrolyze AThTP to ThDP and AMP [17].

### 4.5. AThTP Hydrolysis in Animal Tissues

In animal tissues, AThTP is hydrolyzed by a membrane-bound enzyme probably of microsomal origin [98]. In chicken and rat liver homogenates, this enzyme has a pH optimum of 8.0–8.5, does not require Mg^2+^ as cofactor, and obeys Michaelis–Menten behavior with an apparent K_m_ around 50 µM. The enzyme activity is highest in liver and kidney tissues.

### 4.6. Physiological Roles of Adenylated Thiamine Derivatives

Concerning the dephosphorylated compound AThDP, we only know that it is present in minimal amounts in *E. coli* and mammalian liver and its possible role and metabolism are unknown. The fact that in mammalian tissues it is only detected in the liver, a metabolically highly active organ involved in many detoxification reactions, might suggest that AThDP is a mere byproduct, possibly of the enzyme complex responsible for AThTP synthesis.

The situation is different for AThTP. This compound accumulates in *E. coli* under very specific conditions of energy stress and may reach up to 15% of total thiamine. It is thus possible that it may act as some kind of alarmone. 

Recently, a very interesting hypothesis was suggested: AThTP might form 5′-thiamine-capped RNAs [90]. The authors were able to obtain short 5′-thiamine-capped RNAs with T7 RNA polymerase, but the existence of such caps in bacteria now needs to be proven.

It was reported that AThTP (10 µM) inhibits mammalian poly(ADP-ribose) polymerase-1, an enzyme involved in various stress-related diseases, such as diabetes mellitus [104]. Molecular modeling of the binding of AThTP to poly(ADP-ribose) polymerase-1 suggests a U-shaped conformation of the thiamine vitamer as suggested by ^1^H-NMR studies [96]. It is, however, not known whether such an interaction occurs in vivo. However, inhibition of poly(ADP-ribose) polymerase-1 by AThTP was not reproduced in a later study [99]. 

Just like ThTP, AThTP activated purified GDH at µmolar concentrations [14].

## 5. Conclusions

Sixty years after the discovery of ThTP, its physiological remains frustratingly elusive. A specific role of ThTP in nerve excitability, as first claimed, could not be proven until now [16,23]. However, three major breakthroughs opened new gateways towards the understanding of its role.

1. The first was the discovery that in *E. coli*, ThTP is induced under conditions of amino acid starvation in the presence of a carbon source, leading to the oxidation of pyruvate [28]. These results obtained from *E. coli* cells cannot necessarily be extrapolated to animal cells. Indeed, there are no known conditions where animals reversibly accumulate ThTP (we do not consider here constitutive synthesis by adenylate kinase in skeletal muscle and electric organs due to inactive THTPA).
2. The recognition that, in addition to being a byproduct of AK1, a reaction that is probably physiologically irrelevant, ThTP is synthesized by ATP synthase in *E. coli*, in mammalian mitochondria isolated from brain, and probably plants [29,32,37].
3. The molecular characterization of the soluble 25-kDa ThTPase and its extremely high specificity for ThTP as substrate [41,73].

These data do not favor a role of ThTP in membrane excitability but rather a metabolic role. With this respect, it is intriguing that ThTP (and AThTP) activated purified glutamate dehydrogenase, a mitochondrial enzyme, at physiologically relevant concentrations of 1–10 µM [14]. Hence, the present data suggest that ThTP might play a role at the interface between pyruvate oxidation and amino acid metabolism (Figure 3). This is also in agreement with the observation that in *E. coli*, glutamine synthetase seems to be required for ThTP synthesis in vivo (Figure 2B). 

As for the other protagonist, THTPA, it is difficult to assume that an enzyme with such a high specificity for its substrate ThTP and such a high catalytic efficiency would have a role unrelated to ThTP hydrolysis. The possibility that THTPA is involved in the rescue of the coenzyme ThTP [93] remains a possibility, but it is not very likely as, in the brain for instance, ThTP is synthesized inside mitochondria. An intriguing point is the apparent post-transcriptional regulation of THTPA expression and increased expression of the protein in highly differentiated versus less differentiated cells [26,74,90] with the possible exception of spermatozoa [74]. It is probable that the production of a *thtpa* knockout mouse, provided transmitting chimera can be obtained, will yield precious information concerning the possible physiological role of the ThTP/THTPA couple.

Even more intriguing is the recent discovery of a second triphosphorylated thiamine compound, AThTP. In *E. coli*, AThTP is synthesized under conditions opposite to those leading to ThTP synthesis [17,103], i.e., carbon starvation, membrane potential collapse, suggesting a possible role in response to energy stress.

In conclusion, it is hard to imagine that these high-energy compounds are just an accident of nature, in particular in *E. coli*, where they are synthesized under very specific and highly reproducible conditions of metabolic stress. Though such conditions have not yet been found in mammalian cells, they express a highly specific ThTPase involved in the regulation of cytosolic ThTP concentrations. Hence, thiamine triphosphorylated derivatives still provide a challenging topic for future studies. In particular, the study of the regulation of ThTP and AThTP in *E. coli* might give interesting clues as to the possible roles of these compounds. In animals, the successful production of *thtpa* knockout mice might be of interest. The identification of the AThTP synthesizing and hydrolyzing enzymes combined with transgenic animals might also constitute a promising approach.

## Data Availability

Not applicable.

## Abbreviations

AK1	Adenylate kinase 1 (myokinase)
AThDP	Adenosine thiamine diphosphate
AThTP	Adenosine thiamine triphosphate
GDH	Glutamate dehydrogenase
GS	Glutamine synthetase
ThDP	Thiamine diphosphate
ThMP	Thiamine monophosphate
ThTP	Thiamine triphosphate
THTPA	25-kDa ThTPase
ThTPase	Thiamine triphosphatase
TPK1	Thiamine diphosphokinase 1
TTM	Triphosphate Tunnel Metalloenzyme
